# Sinusoidal Response of dc SQUIDs For rf Power Measurements

**DOI:** 10.6028/jres.092.023

**Published:** 1987-08-01

**Authors:** Robert L. Peterson

**Affiliations:** National Bureau of Standards Boulder, CO 80303

**Keywords:** attenuation, rf-measurements, SQUID, superconductivity

## Abstract

Current, power, and attenuation measurements with rf SQUIDs are based on the fact that the voltage from the microwave readout circuit can be made a nearly sinusoidal function of the magnetic flux threading the SQUID. We point out here that an asymmetric dc SQUID with sufficiently low inductance can achieve a very sinusoidal output voltage with good modulation depth. The spectral purity of the sinusoid can be substantially better than that obtained with rf SQUID systems. The purity improves with increasing asymmetry of the junction critical currents, and decreasing values of the *LI_c_* product, where *L* is the SQUID inductance and *I_c_* is the smaller of the critical currents. Results of several calculations are presented. Substantial improvement in SQUID methods of rf current, power, or attenuation measurement may thus be possible with use of such dc SQUIDs.

## Introduction

In the early 1970s, Kamper et al. [1–5]^1^ recognized that superconducting quantum interference devices (SQUIDs), with their periodic response to magnetic flux Φ, had the potential for being used in a completely new kind of current, power, or attenuation measurement. In the systems developed, the rf impedance of a single-junction SQUID (rf SQUID) was measured. The impedance variations were reflected into a resonant circuit and measured as variations in phase or amplitude of the output rf voltage. If the voltage response is purely sinusoidal in flux with period equal to the flux quantum Φ_o_=*h*/2*e*,

$$V=V_{0}\;\cos\;\left(2\pi \Phi /\Phi_{0}\right),$$ (1)

the time-averaged voltage response to a sinusoidally varying flux of amplitude Φ*_m_* takes the form

$$\bar{V}=V_{0}J_{0}\left(2\pi \Phi_{m}/\Phi_{0}\right).$$ (2)

Equation (1) is achieved approximately with use of an overdamped SQUID and a broadband microwave readout circuit [4]. By determining the zeroes of the response experimentally as the flux amplitude is varied, and equating them to the zeroes of the Bessel function *J*_0_, one can determine Φ*_m_* or its equivalent in terms of current.

**Figure 1:**
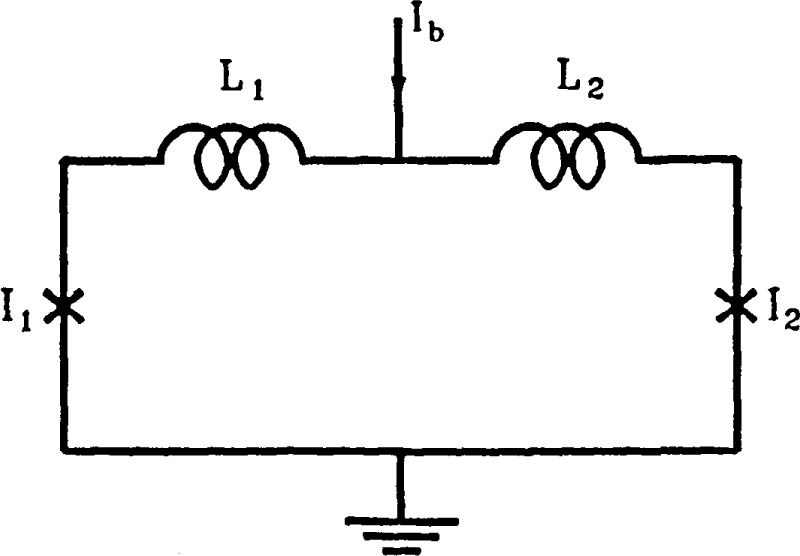
Diagram of a dc SQUID with bias current. The inductances and junctions on the two sides may be different. The crosses represent the junctions, including resistance and capacitance.

The periodic response to an impressed flux is, however, not ideally sinusoidal, and this necessitates an elaborate series of corrective procedures [2–7]. The purpose of this note is to point out that a dc SQUID (a superconducting loop containing two Josephson junctions—see fig. 1) with asymmetric critical currents and sufficiently small inductance and capacitance can have a considerably purer sinusoidal voltage output with adequate modulation. Thus one of the major sources of difficulty in realizing a Josephson junction device for rf measurements can potentially be removed. Although the SQUID inductance is small, the current in the input coil necessary to couple flux into the SQUID can be kept at a reasonable level. The noise in such SQUIDs is also expected to be small.

**Figure 2:**
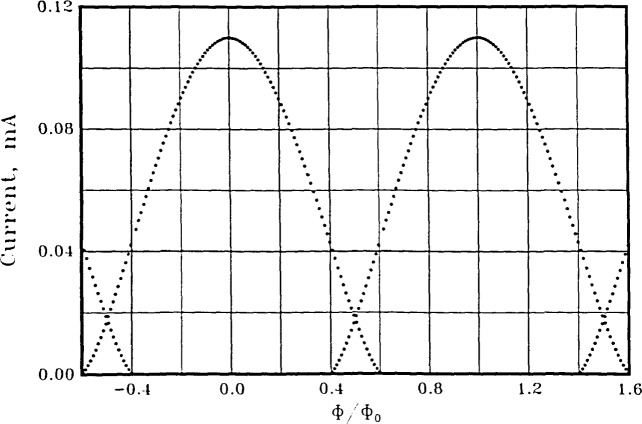
The positive half of the threshold characteristic of a symmetric dc SQUID having *L*_1_=*L*_2_=2.0 pH and *I*_01_*=I*_02_=55 *μ*A.

The *threshold* characteristic (i.e., maximum zero-voltage current *vs*. flux) of a symmetric dc SQUID is not at all sinusoidal. Figure 2 shows an example for a 4-pH SQUID with equal critical currents of 55 *μ*A. It has long been known, however, that the *voltage* developed across a symmetric SQUID has a sinusoidal appearance when the bias current is sufficiently greater than the maximum critical current of the device. This is observed experimentally and from calculations (see, e.g., [8] and references therein).

That an *asymmetric* dc SQUID can exhibit a sinusoidal threshold characteristic has been noted earlier by us [9] as well as by Fulton et al. [10]. No specific application was seen at the time. Intuition suggests, however, that this sinusoidal character should be reflected in the voltage developed across the SQUID, and calculations bear this out, as we shall presently show.

The equations describing the threshold behavior of a dc SQUID are [9]

$$\cos\;\phi_{2}=-\cos\;\phi_{1}/\left(\alpha +\beta \;\cos\;\phi_{1}\right),$$ (3)

$$I_{m}=I_{01}\;\sin\;\phi_{1}+I_{02}\;\sin\;\phi_{2},$$ (4)

$$2\pi \Phi /\Phi_{0}=\phi_{2}-\phi_{1}+\beta_{2}\;\sin\;\phi_{2}-\beta_{1}\;\sin\;\phi_{1},$$ (5)

where *ϕ*_1_ and *ϕ*_2_ are the quantum mechanical phase differences across the two Josephson junctions, *I*_01_ and *I*_02_ are the respective critical currents, *α=I*_Q2_/*I*_01_, *β_i_*=2*πL_i_I*_01_*_i_*/Φ_0_, *β*=2*πLI*_02_./Φ_0_, *L=L*_1_+*L_2_*, and *L*_1_ and *L*_2_ are the lumped inductances for the two segments of the SQUID. See figure 1. Equation (3) shows that there may not be a real solution for *ϕ*_2_ if the denominator on the right is less than unity. In fact if *α* and *β* are each much less than unity, *ϕ*_2_ has a solution only for *ϕ*_1_ very close to (2*n*+1)*π*/2 where *n* is any integer or zero. Choosing *ϕ*_1_≈*π/*2 and noting that the requirements of a ≪ 1 and *β* ≪ 1 requires *β*_2_ ≪ 1 (although *β*_1_ may still be of the order of unity), we find from eqs (4) and (5) that

$$\begin{array}{c} I_{m}\approx I_{01}+I_{02}\;\sin\;\left(2\pi \Phi /\Phi_{0}-\beta_{2}\;\sin\;\phi_{2}+\pi /2\beta_{1}\right)\\ \approx I_{01}+I_{02}\;\cos\;\left(2\pi \Phi /\Phi_{0}-\beta_{1}\right). \end{array}$$ (6)

This establishes the approximate sinusoidal behavior of the threshold characteristic of a sufficiently asymmetric dc SQUID with low inductance.

**Figure 3:**
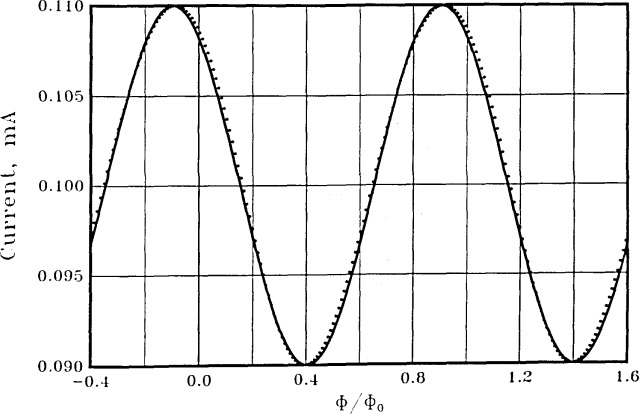
The positive portion of the threshold characteristic of an asymmetric dc SQUID with *L*_1_=*L*_2_=2.0 pH, *I*_01_=100 *μ*A, *I*_02_=10 *μ*A. The solid line is the sinusoid of eq (6) with *β*_1_=0.61.

In figure 3 we show the computed threshold behavior of an asymmetric dc SQUID, having equal inductances *l*_1_=*l*_2_=2.0 pH, and unequal critical currents *I*_01_ = 100 *μ*A, *I*_02_ = 10 *μ*A. We also plot the perfect sinusoid of eq (6) for comparison. Calculations show that the spectral purity of the threshold characteristic can be improved by making the inductances asymmetric as well. This remarkable purity of the threshold characteristic is carried over into the voltage across the SQUID.

The dynamic equations describing a dc SQUID are

$$\begin{array}{l} L_{1}C_{1}\frac{d^{2}\phi_{1}}{dt^{2}}-L_{2}C_{2}\frac{d^{2}\phi_{2}}{dt^{2}}+\frac{L_{1}}{R_{1}}\frac{d\phi_{1}}{dt}-\frac{L_{2}}{R_{2}}\frac{d\phi_{2}}{dt}\\ +\phi_{1}-\phi_{2}+\beta_{1}\;\sin\;\phi_{1}-\beta_{2}\;\sin\;\phi_{2}+2\pi \frac{\Phi }{\Phi_{0}}=0, \end{array}$$ (7)

$$\begin{array}{l} C_{1}\frac{d^{2}\phi_{1}}{dt^{2}}+C_{2}\frac{d^{2}\phi_{2}}{dt^{2}}+\frac{1}{R_{1}}\frac{d\phi_{1}}{dt}+\frac{1}{R_{2}}\frac{d\phi_{2}}{dt}\\ +\frac{\beta_{1}}{L_{1}}\;\sin\;\phi_{1}+\frac{\beta_{2}}{L_{2}}\;\sin\;\phi_{2}=2\pi \frac{I_{b}}{\Phi_{0}}. \end{array}$$ (8)

Here the *C*’s are the junction capacitances and the *R*’s are resistances shunting the junctions. *I_b_* is the bias current injected as shown in figure 1. To calculate the voltage across the SQUID, we solve eqs (7) and (8) with a fourth-order Runge-Kutta technique. We then time-average over typically 10 cycles to obtain the voltages shown in the figures. The precision of our calculations is estimated as a few parts in 10^5^.

**Figure 4:**
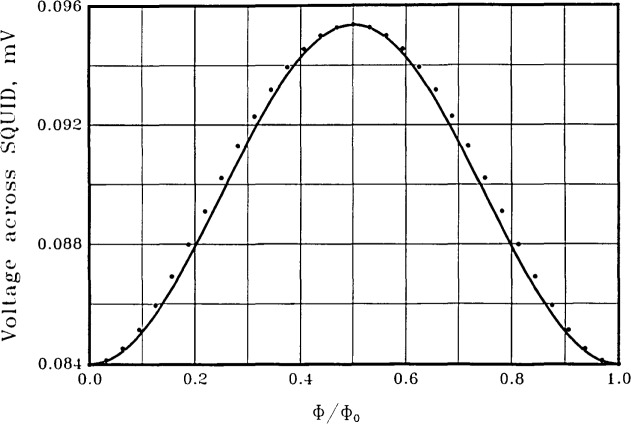
The time-averaged voltage across the symmetric SQUID of figure 2 with a current bias of 200 *μ*A (dots). Shunt resistances of 1 Ω and junction capacitances of 0.66 pF are used. The solid line is a sinusoid for visual comparison.

In figure 4 we show the calculated time-averaged voltage across the symmetric SQUID of figure 2 for a bias current of 200 *μ*A, about twice the maximum critical current A sinusoid is also shown for visual comparison. Shunt resistances of 1 Ω and junction capacitances of 0.66 pF are used for the two equal junctions. The junction capacitances are calculated by assuming a current density of 1000 A/cm^2^, together with a specific capacitance of 12 *μ*F/cm^2^, characteristic of Nb. The voltage modulation at the bias current used is about 11 *μ*V relative to an average voltage of about 90 *μ*V, or about 12 percent modulation.

**Figure 5:**
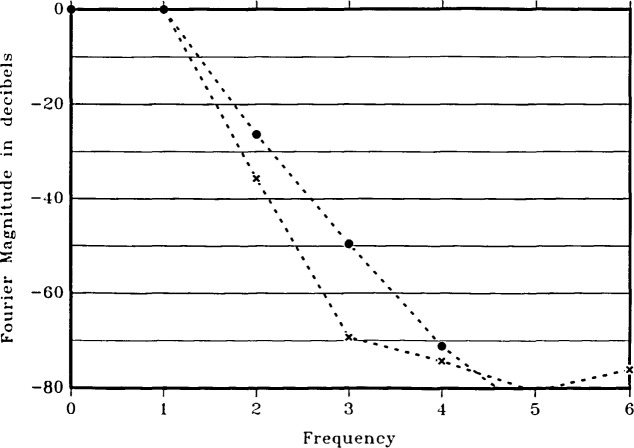
Frequency spectra, in decibels, of the time-averaged voltage across two dc SQUIDs. The solid circles represent the symmetric SQUID of figures 2 and 4. The crosses represent the asymmetric SQUID of figures 3 and 6. The two SQUIDs have the same total inductance and critical current. Levels below about −70 dB are significantly affected by computational noise (see text).

Figure 5 shows the frequency spectra in decibels for this case as well as for an asymmetric case. The dc component is suppressed. The spectra are obtained by calculating 32 voltage points in one period of the flux, then using a 32-point Fast Fourier Transform (FFT). Unity on the horizontal axis represents the fundamental period Φ_0_. The imprecision in the voltage calculations is amplified somewhat in passing through the FFT. Thus, points below about −70 dB in figure 5 are significantly affected by computational noise.

The solid circles in figure 5 show the spectrum of the symmetric SQUID of figure 4. The spectral purity of the voltage of this SQUID is better than that reported for an rf SQUID [4]. The second harmonic is about 26 dB below (5 percent of) the fundamental, and the third harmonic is 50 dB down (0.3 percent).

**Figure 6:**
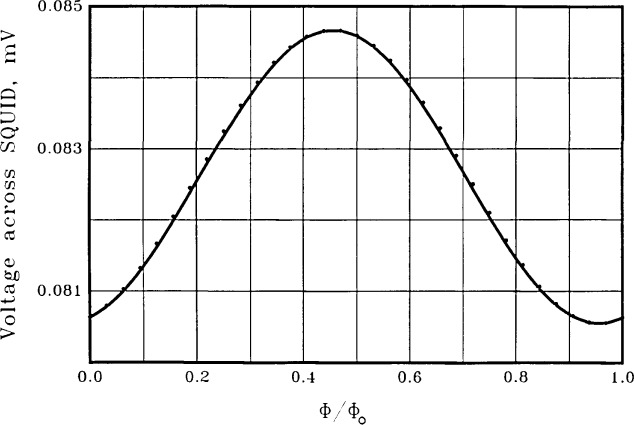
The time-averaged voltage across the asymmetric SQUID of figure 3 with a current bias of 200 *μ*A (dots). Shunt resistances of 1 Ω each are used. Junction capacitances are *C*_1_= 1.2 pF and *C*_2_ = 0.12 pF. The solid line is a sinusoid for visual comparison.

Figure 6 shows the time-averaged voltage *vs*. flux for the asymmetric SQUID of figure 3, again with a bias current of 200 *μ*A and shunt resistances of 1 Ω. The junction capacitances are calculated as above and have values of 1.2 and 0.12 pF, corresponding to critical currents of 100 *μ*A and 10 *μ*A. The calculations show an excellent voltage sinusoid with a voltage modulation of 4 *μ*V relative to an average voltage of about 83 *μ*V, or about 5 percent modulation. A perfect sinusoid is also shown for visual comparison. The frequency spectrum of this SQUID is shown as the crosses in figure 5. The improvement in spectral purity of the asymmetric SQUID over the symmetric SQUID with the same total inductance and critical current is substantial. The second and third harmonics are now down 36 and 69 dB. The depth of modulation of the voltage is not as large as with the symmetric SQUID, however. We find this to be generally true —asymmetric SQUIDs have superior spectral purity but less depth of modulation than symmetric SQUIDs with the same bias current and the same total inductance, critical current, and shunt resistance.

**Figure 7:**
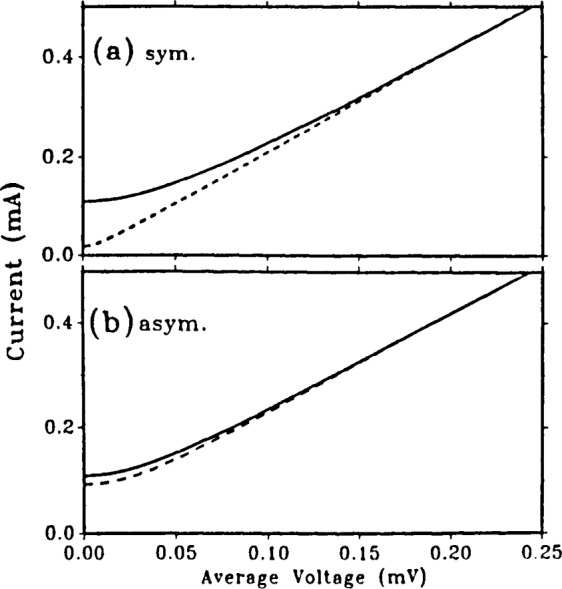
Current-voltage characteristics for dc SQUIDs at Φ=0 (solid curves) and Φ=0.5Φ_0_ (dashed curves). *L*_1_=*L*_2_=2.0 pH and *R*_1_*=R*_2_=1 Ω for both cases, (a) Symmetric SQUID with *I*_01_=*I*_02_=55 *μ*A, *c*_1_=*C*_2_=0.66 pF. (b) Asymmetric SQUID with *I*_01_=100 *μ*A, *I*_02_=10 *μ*A, *C*_1_ = 1.2 pF, *C*_2_=0.12 pF.

**Figure 8:**
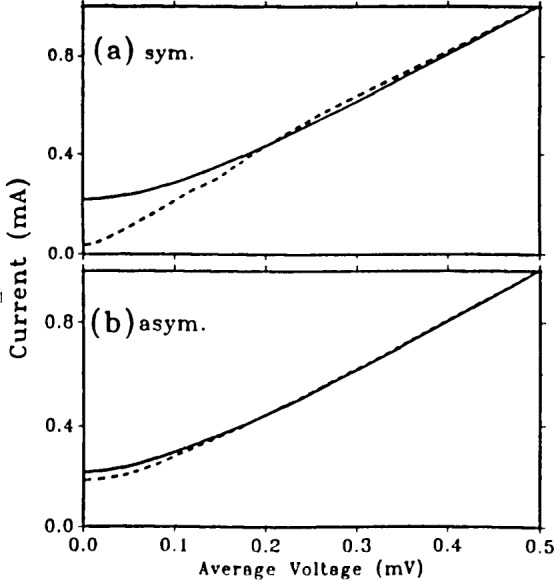
Current-voltage characteristics for dc SQUIDs at Φ=0 (solid curves) and Φ=0.5Φ_0_ (dashed curves). *L*_1_=*L*_2_=1.0 pH and *R*_1_*=R*_2_=1 Ω for both cases, (a) Symmetric SQUID with *I*_01_=*i*_02_=110 *μ*A, *C*_1_=*C*_2_=1.32 pF. (b) Asymmetric SQUID with *I*_01_=200 *μ*A, *I*_02_=20 *μ*A, *C*_1_=2.4 pF, *C*_2_=0.24 pF.

The computed I–V curves of figures 7(a,b) show the differences in modulation for the two SQUIDs considered here. Curves are shown for Φ=0 and Φ=0.5Φ_0_. Note that these values of flux are not quite at the extrema of the voltage for the asymmetric SQUID, as figure 6 shows. Spectral purity generally increases as the bias current is increased, but at the expense of depth of modulation. An exception occurs when an LC resonance significantly affects the current-voltage relation, as shown in figure 8. Here the shunt resistances are kept at 1 Ω but the critical currents, and hence capacitances, of the SQUIDs considered above are doubled, and the inductances are halved to keep the *LI*_c_ product unchanged. For the symmetric SQUID—figure 8(a)—an LC resonance at $\Phi_{0}/\left(2\pi \sqrt{L_{1}C_{1}}\right)=0.29$ is evident. (The resonance becomes sharper with larger values of shunt resistance.) Calculations for SQUIDs with apparent LC resonances show that the spectral purity of the voltage *vs*. flux curves is degraded. The asymmetric SQUID of figure 8(b) does not show a prominent LC resonance because the two sides of the SQUID interfere; however, the larger capacitance of this example greatly reduces the modulation depth. It is thus important that the junction capacitance be kept as low as possible so that the capacitive impedance does not shunt out the resistance.

Perhaps the principal virtue of the low-inductance SQUIDs, and especially the asymmetric low-inductance SQUIDs, lies in the fact that the harmonics above the second are very greatly reduced and are probably negligible. This is important because the earlier work with rf SQUIDs [5] found that the second harmonic could effectively be nulled, and that the higher harmonics constituted the principal problem. That the second harmonic is also substantially reduced is of course an improvement. Since the basic source of systematic error in the measurement of rf attenuation is caused by harmonic distortion of the sinusoidal response of the system [4], the SQUIDs discussed here should have a distinct advantage over the rf SQUIDs.

In 1982 M. Cromar of this laboratory made a preliminary study on the suitability of a thin-film dc SQUID as the detector element in an rf attenuator-calibrator system (unpublished). Using a resistively shunted symmetric SQUID whose *LI*_c_ product was considerably larger than Φ_0_, he showed that at low signal frequencies, the zeroes of the response approximated the zeroes of the *J*_0_ Bessel function closely enough that accuracy at rf frequencies comparable to that of the earlier measurements with rf SQUIDs might be realized. This research was not pushed further.

The microfabricated dc SQUIDs are expected to have better thermal and temporal stability than the single-junction rf SQUIDs used earlier. The overall circuitry necessary to attain the output voltage should also be simpler with the dc SQUID. Imperfections in microwave components were found to be a major contributor to the harmonic distortion in the rf SQUID system [5]. Such components would not be used in the readout scheme for a dc SQUID.

The 4-pH inductances used here are small, but SQUIDs with lower inductance have been fabricated. The low inductance of the proposed SQUIDs presents a potential problem of sufficient coupling, however. For example, if the mutual inductance between a 4-pH SQUID and the input line were only 4-pH, about 500 *μ*a would be required in the line to produce one flux quantum in the SQUID. If one desires 200 nulls in the voltage response (100 flux quanta), about 50 mA must flow in the line in this case. Although higher than typical, this value is still below what a superconducting stripline could support. Higher values of mutual inductance will decrease the maximum current needed. The mutual inductance to an input coil carrying the signal can in fact be made much larger than the self-inductance of the SQUID. Planar coupling to low-noise, low-inductance SQUIDs is the subject of much current research [11–13]. Of course, one may also choose to work with higher- inductance SQUIDs, accepting somewhat less spectral purity if the critical currents are kept at the same values, in order to decrease the maximum current needed in the stripline or to decrease the degree of coupling to the SQUID.

The effect of noise is another consideration. It is beyond the scope of this note to undertake a detailed study of noise effects, which depend upon the readout method used. Ideally, of course, it is desirable that the noise of the SQUID plus its readout system be dominated by the intrinsic noise of the SQUID. Readout schemes with this in view are under active investigation; Ketchen [11] discusses several of them. For a readout system operating at a frequency of about 100 kHz, 1/*f* noise is not a consideration. However, even at lower frequencies where 1/*f* noise dominates, a new readout scheme [14] for significantly reducing the noise has been developed.

Properly fabricated dc SQUIDs have the lowest noise figures of any devices [11]. White noise decreases with decreasing inductance, which is favorable for the SQUIDs under consideration. The parameter 2*πkT/I*_0_Φ_0_ is sometimes used to characterize the noise. If we take *I*_0_=0.11 mA, which is the average of the critical currents used in the preceding examples, we find the value 0.002 at 4 K for this noise parameter. The ultra-low-noise SQUIDs operate near this value. The spectral density *Sv* of the voltage fluctuations in a resistor R is given by 4*kTR*. For shunt resistances of about 1 Ω, the voltage noise power spectrum is 2×10^−22^ *V*^2^/Hz at 4 K. The flux noise density *S*_Φ_ is approximately *S_v_*/|∂V/∂Φ|^2^. The ultra-low-noise SQUIDs are built to be biased at that value of flux that gives the greatest energy sensitivity, which occurs at the largest value of |∂V/∂Φ|. However, in the present case the flux will be swept over many periods of Φ_0_ so that each value of flux contributes almost equally. Since the voltage is nearly sinusoidal, |∂V/∂Φ|^2^ varies almost as sine-squared, which we may replace by 1/2 times an amplitude, to a good approximation. Thus $S_{\Phi }\approx 2kTR\Phi_{0}^{2}/{\left(\pi V_{0}\right)}^{2}$, where *V*_0_ is the voltage amplitude. For the symmetric SQUID of figure 4, *V*_0_≈5.5 *μ*V so that $S_{\Phi }\approx 10^{-13}\Phi_{0}^{2}/\text{Hz}$. These values are not far from values quoted for low-noise SQUIDs [13–15]. Other expressions for the voltage noise power spectrum are available [14, 15] which take into account noise contributions from the circulating current in the SQUID. The noise values from these expressions are not significantly different from those calculated above because of the very small inductance. Although these arguments do not establish that noise will not be a significant problem affecting the accuracy of determining the voltage nulls in rf measurements, they show that the SQUIDs proposed here share characteristics of low-noise SQUIDs, and thus are encouraging.

The purpose of this note has been to suggest a new scheme for rf measurements using SQUIDs, and to demonstrate its potential advantages. A more extensive analysis of feasibility could include the following: simulations of the effect of noise upon the accuracy with which the zeroes of the response can be determined; studies of the magnitude and effects of the parasitic inductance associated with the shunt resistors; inclusion of the circuit carrying the signal—source, coupling coil, and load—to determine whether the nonlinear impedance reflected into the circuit by the SQUID is important; determination of the optimum choice of inductance, critical currents, asymmetry, and bias current to obtain the best combination of spectral purity and depth of modulation, or in short the greatest accuracy possible.

The considerations presented here suggest that thin-film, low inductance, low critical current, dc SQUIDs, especially those with asymmetric critical currents, would be superior to rf SQUIDs in rf current, power, or attenuation measurements.

## Footnotes

1 Numbers in brackets indicate literature references.
